# Diffraction and Polarization Properties of Electrically–Tunable Nematic Liquid Crystal Grating

**DOI:** 10.3390/polym12091929

**Published:** 2020-08-26

**Authors:** Shuan-Yu Huang, Bing-Yau Huang, Chi-Chung Kang, Chie-Tong Kuo

**Affiliations:** 1 Department of Optometry, Chung Shan Medical University, Taichung 402, Taiwan; syhuang@csmu.edu.tw; 2 Department of Ophthalmology, Chung Shan Medical University Hospital, Taichung 402, Taiwan; 3 Department of Physics, National Sun Yat-sen University, Kaohsiung 804, Taiwan; flyfishss31@gmail.com (B.-Y.H.); Kangchi@ymail.com (C.-C.K.); 4 Department of Optometry, Shu-Zen Junior College of Medicine and Management, Kaohsiung 821, Taiwan

**Keywords:** diffraction, polarization, nematic liquid crystal, grating, the first-order diffraction

## Abstract

This work demonstrates an electrically-tunable nematic liquid crystal (NLC) diffraction grating with a periodic electrode structure, and discusses the polarization properties of its diffraction. The efficiency of the first-order diffraction can be gradually controlled by applying external electric fields cross the NLC, and the maximum diffraction efficiency of the first-order diffraction that can be obtained is around 12.5% under the applied voltage of 5.0 V. In addition to the applied electric field, the efficiency of the first-order diffraction can also vary by changing the polarized state of the incident beam. Antisymmetric polarization states with symmetrical intensities in the diffractions corresponding to the +1 and −1 order diffraction signals are also demonstrated.

## 1. Introduction

Polarization is an interesting feature in optics [1], and numerous applications based on the control of polarization have attracted attention recently, such as 3D displays and virtual reality/augmented reality (VR/AR) [2,3,4]. Polarization of light can be manipulated by anisotropic absorption, anisotropic reflection, birefringence materials, or metamaterials; for example, a transverse-magnetic (TM) wave or polarization can be picked out by means of the Brewster angle, while linear polarization can be rotated by a specific angle after passing through an optical active medium. Liquid crystal is a birefringent material that is powerful enough for the modulation of polarization. With the features of controllable birefringence and refractive indices, liquid crystal has been applied in various fields, such as displays [5], ophthalmic optics [6,7,8], beam shaping [9,10,11], and bio-sensors [12,13,14].

Diffraction grating is a dispersion element widely applied in optical systems, and its main applications include spectrometers [15], wavelength division multiplexing (WDM) [16], and external-cavity lasers [17]. As diffraction grating is a key component in optical systems, researchers are devoted to developing tunable diffraction gratings. Studies regarding tunable gratings, as based on liquid-crystal-related materials, have also been extensively proposed; for example, tunable amplitude gratings based on polymer-dispersed liquid crystals [18], optically and electrically controllable gratings based on dye-doped nematic liquid crystals [19,20], tunable phase gratings on the basis of the photo-alignment technique [21], and circular-polarization selective gratings based on cholesteric liquid crystals [22]. Previous literature also shows that the diffraction efficiency of first order diffraction in a dye-doped liquid crystal grating can be influenced by the amount of the doped azo-dyes [20]. Considering the interesting physics and potential applications, liquid crystal grating is a research topic worthy of further investigation.

This paper demonstrates an electrically-tunable diffraction grating, as based on nematic liquid crystals (NLCs) in a specialized cell with periodic electrodes, and discusses the polarization properties of the ±1st order diffractions. The intensities of the diffractions, as well as the variations of the refractive indices, can be controlled by an external electric field via the reorientations of the NLCs. The maximum diffraction efficiency of the 1st order diffraction can be 12.5% when the applied voltage is 5.0 V. The experimental results also indicate that the polarization states of the ±1st order diffractions deviate from the initial polarization state by 15° due to the tilting and azimuthal rotations of the NLCs in the edge regions of the electrodes.

## 2. Materials and Methods

**Figure 1:**
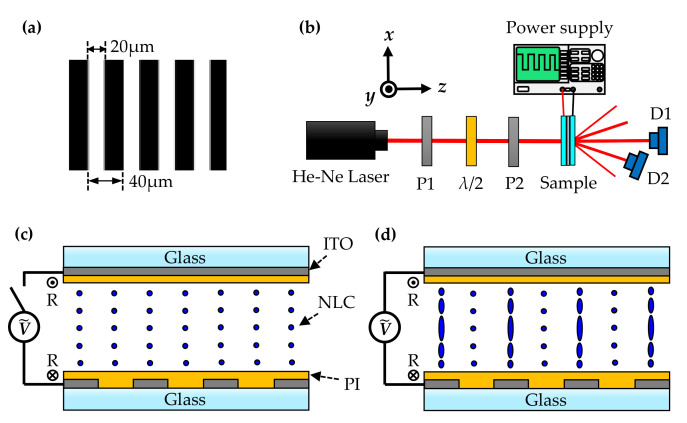
Schematics of (**a**) the electrodes, (**b**) the experimental setup, (**c**) sample without applied voltage, and (**d**) sample with applied voltage.

Nematic liquid crystal (E7, from Merck, Darmstadt, Germany) was injected into an empty cell with grating-like electrodes for the fabrication of electrically tunable diffraction grating. The empty cell was composed of a glass substrate with grating-like electrodes and an ITO-coated glass substrate with two 38-μm-thick Mylar spacers. The width and space of the grating-like electrodes were 20 and 40 μm, respectively, as shown in Figure 1a. Both the inner sides of the substrates were coated with polyimide (SE-130, from Nissan, Tokyo, Japan) and anti-parallel rubbed by a fabric textile for the alignment of the liquid crystal molecules.

The experimental setup for probing the diffraction of the tunable liquid crystal grating is shown in Figure 1b, where a He-Ne laser with a pair of polarizers and a half-wave plate were aligned as the probe beam. The intensity of the probe beam can be adjusted by properly setting the first polarizer (P1) and the half-wave plate, while the polarization of the probe beam can be controlled via the second polarizer (P2). An alternative-current (AC) field with the frequency of 1 kHz was applied on the sample to trigger the reorientations of the liquid crystal molecules, which formed the diffraction grating. To measure the diffraction efficiency, two photo detectors (ET-2040, from EOT, Traverse, MI, US) were set behind the sample to receive the zeroth order and first order signals, respectively. According to the coordinates provided in Figure 1b, it should be noted that the direction of the grating vector was set along the *x*-axis, while the initial orientation of liquid crystal molecules was along the *y*-axis. The polarization of the probe beam was set to be parallel to the *y*-axis. To investigate the polarization properties of the ±1st order diffractions, the photo detectors were set to receive the signals of the ±1st order diffractions, and an additional analyzer was placed between the sample and the photo detectors. In order to analyze the intensities of all the electrically tunable diffractions, the photo detectors were replaced by a lens and a CCD camera.

## 3. Results

**Figure 2:**
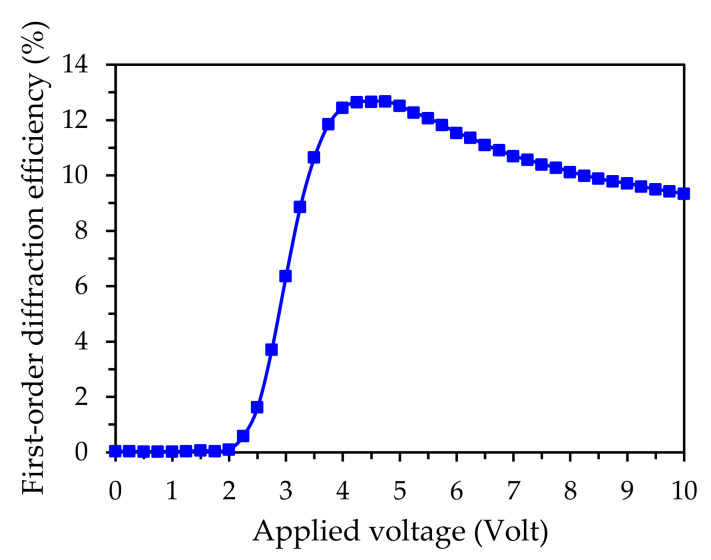
Effect of the applied voltage variation on the first-order diffraction efficiency of the electrically tunable grating.

The first order diffraction efficiencies are recorded as a function of the applied voltage, as shown in Figure 2. When the applied voltage is below 2.5 V, as all the NLCs are aligned parallel to the direction of the probe polarization, no diffraction can be measured. When the applied voltage is 2.5 V, the LC molecules start to become slightly reoriented by the electric field, thus, the difference between the refractive indices at the electrode and non-electrode regions is initiated. However, as the difference between the refractive indices at electrode and non-electrode regions is not large, the diffraction phenomenon is not obvious. As the voltage is increased to 5.0 V, the electric field significantly drives the LC molecules in the electrode regions, which results in a gradient of the refractive index around the periodic electrode structures; thus, the maximum value of the first order diffraction efficiency that can be obtained is ~12.5%. As the voltage exceeds 5.0 V, the LC molecules in the electrode regions are almost reoriented to the direction of the *z*-axis by the electric field. However, as the electric field is also large enough to reorient the LC molecules outside the electrode regions, it leads to the decreased difference of the refractive index around the electrode stripes boundary. The decreased difference in the refractive index (Δ*n*), as perceived by the y-linearly polarized light, will gradually diminish the diffraction phenomenon.

This study defines the diffraction efficiency as the ratio of the first-order diffraction intensity to the zeroth-order diffraction intensity, as represented by Equation (1),

(1) $$\eta =\frac{I_{1}}{I_{0}}$$

where *I*_0,_ and *I*_1_ are the intensities of the zeroth- and the first-order diffraction, respectively. The diffraction efficiency can be calculated by Equation (2) [23],

(2) $$\eta =\sin^{2}\frac{\pi \Delta nd}{\lambda \cos\beta }$$

where *λ* and *β* are the wavelength and the incident angle of the probe beam, respectively; *d* is the thickness of the liquid crystal layer; and Δ*n* is the difference of the refractive indices in the grating. For the case of the sample operated at 5.0 V, the diffraction efficiency *η* is 12.5%. If we substitute *η* = 12.5%, *d* = 38 μm, *λ* = 0.633 μm, and *β* = 0° into Equation (2), Δ*n* is estimated ~0.0019 [20].

**Figure 3:**
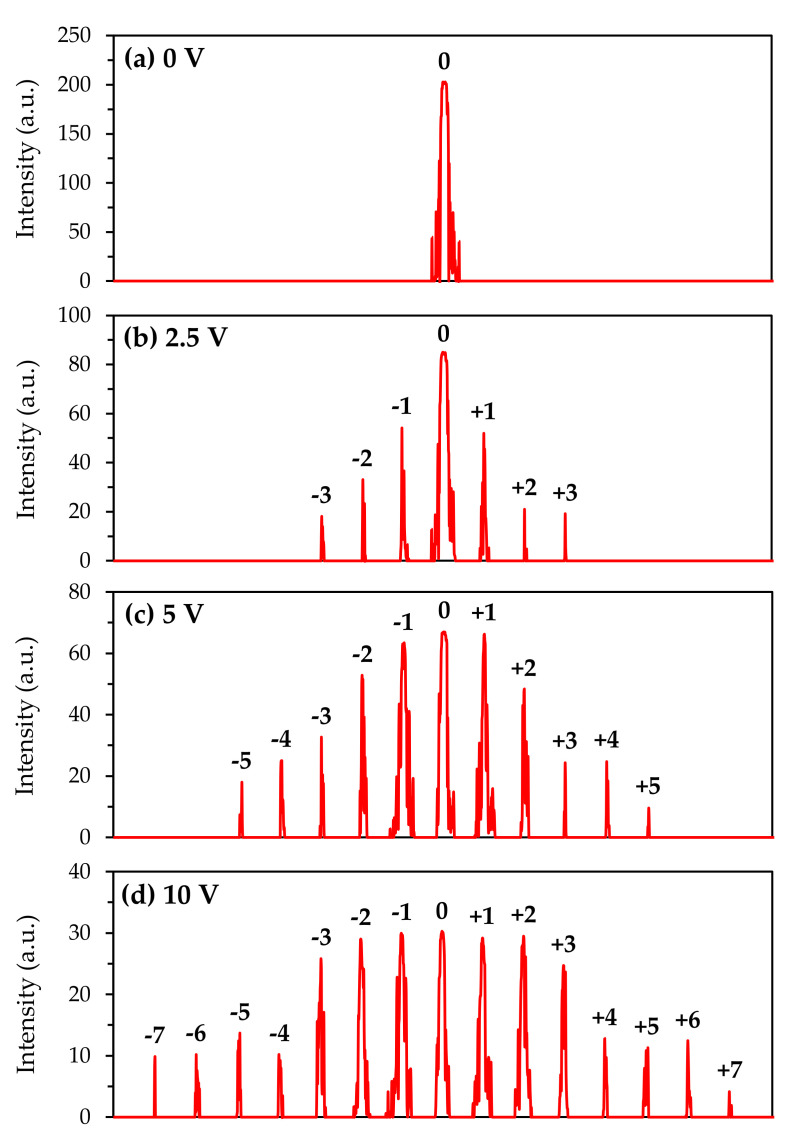
Spatial distributions of the diffraction intensities of the electrically tunable grating at (**a**) 0 V, (**b**) 2.5 V, (**c**) 5.0 V, and (**d**) 10.0 V, respectively.

Figure 3 presents the distributions of the multi-order diffraction intensities with the applied voltage from 0 to 10.0 V. When the voltage is 0 V, as the homogenous alignment of liquid crystal molecules occurs in both electrode and non-electrode regions, the diffraction cannot be observed, thus, only the zeroth-order diffraction (transmitted light) can be measured. When the applied voltage is ~2.5 V, the first-order diffraction appears. As the applied voltage increases to 5.0 V, the 5th diffracted intensity can be detected, and the intensity of the 1st order diffraction is quite close to the zeroth-order diffraction. In this case, the intensity of the 1st order diffraction is the highest among all the diffracted beams. As the applied voltage is ~10.0 V, the intensity of the incident beam will be redistributed to seven pairs of diffractions, and the strengths of the 0th, 1st, and 2nd intensities are almost identical.

**Figure 4:**
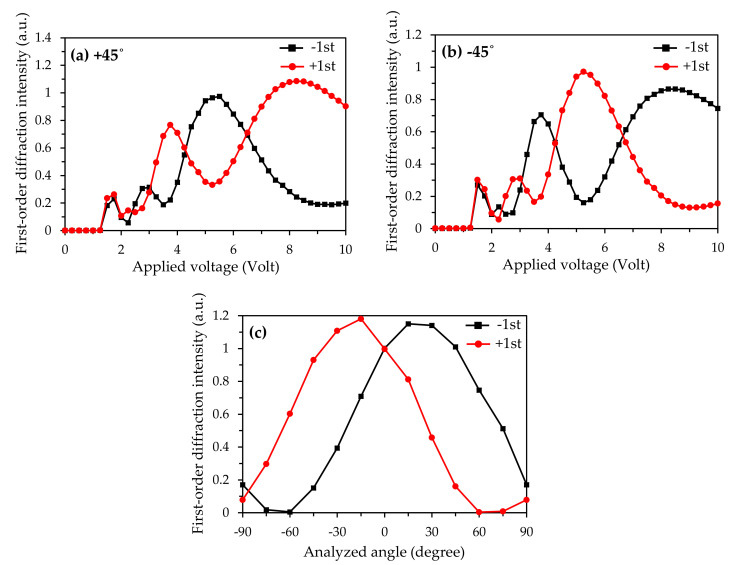
Variations of the applied voltage on the intensities of the ±1st order diffractions when the analyzer is (**a**) 45° and (**b**) −45° relative to the incident polarization. (**c**) Variations of the analyzed angle on the intensities of the ±1st order diffractions.

When the voltage is applied to the sample, the liquid crystal molecules will reorient following the distributions of the electric field between the electrodes inside the sample. The reorientations of the liquid crystal molecules near the edges of the grating-like electrodes and the ITO-coated glass substrate will be different, and will form an electrically induced hybrid twisted nematic configuration [24]. This configuration will sequentially change the polarization states of the incident beam, as well as the diffractions. To analyze the polarization feature of the tunable diffraction grating, this study placed an analyzer between the sample and the photodetectors. Figure 4a,b present the diffraction properties of the ±1st orders when the analyzer is set at +45° and −45° relative to the direction of the incident polarization (y-axis), respectively. When the applied voltage is below 2.0 V, regardless of the configuration of the analyzer (+45° or −45°), the intensities of the ±1st order diffractions are almost identical. When the applied voltage exceeds 2.0 V, the diffraction properties of light intensities in the +1st and −1st orders are asymmetric, and the light intensities of the 1st orders in the +45° polarizer configuration are similar to those of the −1st orders in the −45° polarizer configuration. This result implies that the polarization states of the ±1st order diffractions are antisymmetric. To further check the polarization states of the ±1st order diffractions, the analyzer is rotated under the applied voltage of 5.0 V, as shown in Figure 4c. We can easily find that the maximum intensities of the ±1st order diffractions occur when the analyzer is rotated by 15° counterclockwise and clockwise, respectively. Therefore, when the applied voltage is 5.0 V, the polarizations of the positive and negative first diffractions of this tunable grating have been rotated by −15° and 15°, respectively. 

## 4. Conclusions

In summary, the diffraction and polarization properties of the nematic liquid crystal filled tunable grating cell was investigated in this study. The first order diffraction efficiency was electrically tuned, and the maximum first-order diffraction efficiency of ~12.5% was obtained with the applied external voltage of 5.0 V. The intensity of the first diffraction was also tuned by rotating the polarizer from 0° to 90°. The polarization states of the +1st and −1st were antisymmetric. By properly adjusting the applied voltage, the polarization of diffractions in this grating is expected to be electrically controlled.
